# Necrosis, with Spontaneous Resection of Lower Maxilla

**Published:** 1872-03

**Authors:** Ely Van De Warker

**Affiliations:** Syracuse, New York


### NECROSIS, WITH SPONTANEOUS RESECTION OF LOWER MAXILLA.

By Ely Van De Warker, M. D., Syracuse, New York.

The article of Dr. Whitehead, in the New York Medical Journal, for June, of the current year, recalls to mind the notes of the following case, which I deem important as bearing broadly upon several questions.

It demonstrates clearly the great reserve of *vis conservatrix naturæ* latent in organisms seriously impaired by disease. It is important in its relations to the question of the osteogenetic power of the periosteum; or to the osteogenesis of bone tissue proper; and, lastly, of the disastrous sequelæ of scarlatina. At the time I saw the case it was impossible to define the exact seat of the original inflammatory action. If it were the result of periostitis it is hardly possible to suppose, that, after having sustained such an impairment of vitality as to conduce to the destruction of the subjacent bone, it could still possess sufficient osteogenetic energy to reproduce such an extensive osseous shaft.

On the contrary, if the periosteum participated with the bone in the destructive efforts of the inflammation, the repair must have been either the result of the osteogenetic power residing in each extremity of the divided jaw, and the consequent proliforation of ossific material toward the centre; or, as in the specimen of fracture in the possession of Dr. Dunster, of New York, it must have been the result of ossific developement in the muscular and connective tissue in the neighborhood of the organic mischief. In this view of the subject we must suppose the existence of a remarkable formative power. A beautiful example of the reciprocity of repairative force residing in heterogeneous tissues.

The case is a forcible illustration of the often-times unfortunate sequence of scarlatina. The constitution is seriously impaired; the forces of assimilation are checked, and lurking scrofulous tendencies are developed, which are manifested in glandular affections during adolescence, and culminate in tubercle at puberty.

Case*.—On the 14th of July 1862, I was called to the house of Mrs. L——, widow, to see a little girl whom the messenger informed me, "had something the matter with her mouth." Reaching the house, I found a child between six and seven years of age, with considerable deformity of the lower jaw, on the right side, with inflammatory swelling of the face upon the affected side. The deformity of the jaw presented a smooth, regular outline, and conveyed to the touch the sensation of an osseous tumor.

Inserting my finger into the mouth, I found the right lateral incisior tooth, projecting above the line of the incisior teeth, loose, and between it and the incisiors I could feel the end of a necrosed piece of bone. The fragment of bone appeared so loose, I determined to remove it at once, and if not able to do so, to extract the tooth. I furnished myself with a curved pair of dental forceps, and laid hold of the exposed bone. I drew upon it, but exerted hardly any force. Need I say I was astonished, when I saw almost the entire horizontal portion of the jaw following my forceps?

The operation was attended with but little pain and no hemorrhage.

The necrosed portion of bone measured one-fifth of an inch in length, and was a complete section of the jaw, containing four teeth. The right lateral incisior with the socket of its permanent successor exposed; the canine, the first molar, and the second molar on a line with the alveolar process and in a forward state of development. One feature in the development of the teeth is singular; the right lateral incisior and the second molar seem to be in the same stage of development. The cancellated structure is denuded of its dense external layer, except in that portion round the mental foramen. After the removal of the fragment, I could feel, with the finger in the mouth, a ramus of bone curving downward, causing the deformity, and which united the two divided extremities of bone. In fact, a complete new jaw had been formed, while the necrosed bone was still *in situ*, the new bone having made a bold curve downward to avoid it. There was inability to open the mouth to its full extent, and immobility was the worst distressing symptoms for the three months, during which time I kept the case in view.

---

* The specimen is now in my possession.

